# A Novel *Endo*-Hydrogenase Activity Recycles Hydrogen Produced by Nitrogen Fixation

**DOI:** 10.1371/journal.pone.0004695

**Published:** 2009-03-11

**Authors:** Gordon Ng, Curtis G. S. Tom, Angela S. Park, Lounis Zenad, Robert A. Ludwig

**Affiliations:** Department of Molecular, Cellular and Developmental Biology, Sinsheimer Laboratories, University of California Santa Cruz, Santa Cruz, California, United States of America; University of Liverpool, United Kingdom

## Abstract

**Background:**

Nitrogen (N_2_) fixation also yields hydrogen (H_2_) at 1∶1 stoichiometric amounts. In aerobic diazotrophic (able to grow on N_2_ as sole N-source) bacteria, orthodox respiratory *hupSL*-encoded hydrogenase activity, associated with the cell membrane but facing the periplasm (*exo*-hydrogenase), has nevertheless been presumed responsible for recycling such endogenous hydrogen.

**Methods and Findings:**

As shown here, for *Azorhizobium caulinodans* diazotrophic cultures open to the atmosphere, *exo*-hydrogenase activity is of no consequence to hydrogen recycling. In a bioinformatic analysis, a novel seven-gene *A. caulinodans hyq* cluster encoding an integral-membrane, group-4, Ni,Fe-hydrogenase with homology to respiratory complex I (NADH : quinone dehydrogenase) was identified. By analogy, Hyq hydrogenase is also integral to the cell membrane, but its active site faces the cytoplasm (*endo*-hydrogenase). An *A. caulinodans* in-frame *hyq* operon deletion mutant, constructed by “crossover PCR”, showed markedly decreased growth rates in diazotrophic cultures; normal growth was restored with added ammonium—as expected of an H_2_-recycling mutant phenotype. Using *A. caulinodans hyq* merodiploid strains expressing β-glucuronidase as promoter-reporter, the *hyq* operon proved strongly and specifically induced in diazotrophic culture; as well, *hyq* operon induction required the NIFA transcriptional activator. Therefore, the *hyq* operon is constituent of the *nif* regulon.

**Conclusions:**

Representative of aerobic N_2_-fixing and H_2_-recycling α-proteobacteria, *A. caulinodans* possesses two respiratory Ni,Fe-hydrogenases: HupSL *exo*-hydrogenase activity drives exogenous H_2_ respiration, and Hyq *endo*-hydrogenase activity recycles endogenous H_2_, specifically that produced by N_2_ fixation. To benefit human civilization, H_2_ has generated considerable interest as potential renewable energy source as its makings are ubiquitous and its combustion yields no greenhouse gases. As such, the reversible, group-4 Ni,Fe-hydrogenases, such as the *A. caulinodans* Hyq *endo*-hydrogenase, offer promise as biocatalytic agents for H_2_ production and/or consumption.

## Introduction

*Azorhizobium caulinodans* is an obligate oxidative, microaerophilic bacterium originally isolated from stem- and root-nodules of the legume host plant *Sesbania rostrata* [1]. In legume nodules, endosymbiotic rhizobia, including *A. caulinodans*, fix atmospheric dinitrogen (N_2_) yielding ammonium as utilizable N-source for the host plant. Unlike typical rhizobia which fix N_2_ only endosymbiotically, *A. caulinodans* is also diazotrophic (able to grow on N_2_ as N-source in pure culture). Both processes are owed to molybdenum-containing (Mo) dinitrogenase, an α_2_β_2_-tetrameric protein complex catalyzing directed electron-transfer. Metabolic electrons are tapped from pyruvate oxidation [2] and singly transmitted via flavo- and FeS-proteins ultimately to the Mo-dinitrogenase catalytic center, its iron-molybdenum cofactor (FeMo-co); the enzyme complex effectively operates an 8-electron reductive cycle of recursive single electron transfers [3], [4]. At the FeMo-co center, the first two arriving electrons combine with hydrogen-ions to yield a molecule of H_2_. The bound H_2_ is then displaced by N_2_, and the subsequent six, arriving electrons, together with hydrogen-ions, now reduce N_2_ to yield two molecules of ammonium as co-product:
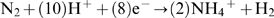



*In vivo*, H_2_ yields (relative to 1∶1 *in vitro* stoichiometry) may further increase as a function of Mo-dinitrogenase turnover [5]. As the substrate N_2_ triple-bond is highly unreactive, the dinitrogenase catalytic cycle is kinetically limiting as an *in vivo* biochemical standard process. To accelerate catalysis and render such thermodynamically favorable, Mo-dinitrogenase is both one-electron reduced and energetically charged by homodimeric dinitrogenase reductase, which harbors a bridging 4Fe-4S-center and two ATP binding sites, one per subunit. During single-electron transfer from dinitrogenase reductase to Mo-dinitrogenase, 2 ATP hydrolyze to yield 2 ADP and 2 orthophosphate (Pi). Thus, in the 8-electron dinitrogenase complex catalytic cycle:
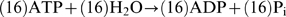
earning Mo-dinitrogenase complex activity distinction as the most ATP-consumptive metabolic reaction on a per substrate basis [5].

Notably, *A. caulinodans* diazotrophic cultures, as with other aerobic diazotrophic bacteria, do not evolve significant H_2_. Rather, H_2_ produced by Mo-dinitrogenase complex activity is efficiently recycled as respiratory electron donor to O_2_ (as preferred electron-acceptor), thus recouping by oxidative phosphorylation ATP invested in H_2_ production as part of the dinitrogenase catalytic cycle:
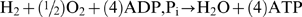
which represents some 25% of total ATP invested in N_2_ fixation [6].

H_2_ production has long been associated with N_2_ fixation in pure diazotrophic cultures of both fermentative and oxidative bacteria as well as by endosymbiotic rhizobia in legume nodules [7]. Endogenous H_2_ recycling, both in diazotrophic bacterial cultures [8] as well as in certain symbiotic nodules, among those, garden pea [9], has been presumed owed to a respiratory Ni,Fe-hydrogenase activity highly conserved among disparate aerobic diazotrophic bacteria [10], [11]. As studied in archetypal aerobic bacteria such as *Ralstonia eutropha*, orthodox respiratory hydrogenase is a heterodimeric protein comprising a bimetallic Ni,Fe-catalytic subunit and a 4Fe-4S-center subunit, which complex with an integral-membrane *b*-type cytochrome, linking Ni,Fe-hydrogenase H_2_-oxidizing activity to cellular respiration and oxidative phosphorylation [12].

To the contrary, as we report here for *A. caulinodans* diazotrophic cultures open to the environment, endogenous H_2_ is not recycled via orthodox respiratory *exo*-hydrogenase activity but instead via a novel respiratory *endo*-hydrogenase complex, presumably reflecting the need to sequester endogenous H_2_ by metabolic channeling.

## Results

### *A. caulinodans exo*-hydrogenase deletion mutants lose chemoautotrophy but retain diazotrophy

**Figure 1:**
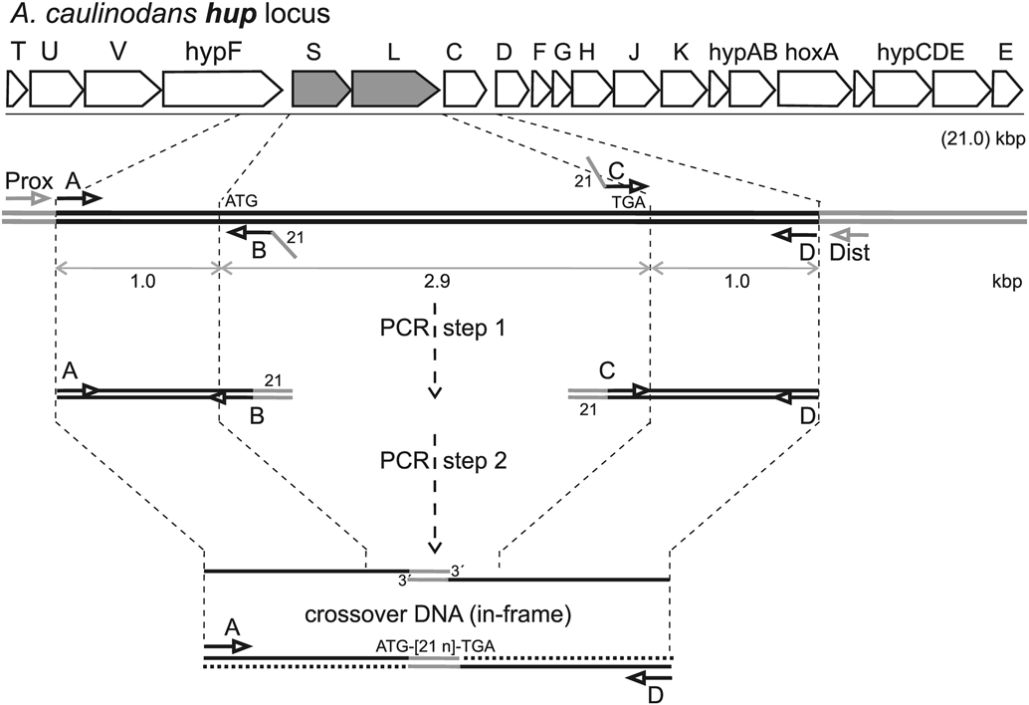
*A. caulinodans hup* genetic locus and physical map: creation of in-frame translational fusion deletions. The top line represents the genetic map of the 20-gene *hup* polycistronic operon spanning 21 kbp. The second line represents an expanded physical map of *hupSL* DNA indicating postions of and (5′→3′) polarity for synthetic A, B, C, and D oligodeoxynucleotide primers of genome-identical sequence used in two, separate PCR reactions to generate DNA fragments A→B and C→D. The third line indicates a follow-up PCR reaction in which DNA fragments A→B and C→D were mixed, thermally denatured, allowed to partially renature, and used as combination PCR template/primer. As synthetic primers B and C share a complementary 21 bp extension sequence (angled line), the A→B Watson and C→D Crick strands (and *vice versa*) may partially reanneal via this 21 bp linker sequence In the third line, when such occurs, the resulting, partially-reannealed A→B(21 bp)C→D spliced DNA fragment which carries 5′-overhangs and free 3′-ends on both strands is a template for the thermostable DNA polymerase elongation reaction, producing a finished A→D duplex DNA fragment which may then be further amplified by PCR in the presence of added A and D primers. As verified by DNA sequencing analysis, finished, amplified A→D duplex fragments carry a genetic crossover which fuses (via the 21 bp linker sequence) in-frame the “start” codon of the proximal *hupS* gene to the “stop” codon of the distal *hupL* gene. Primers A and D may be extended with genome non-complementary elements to facilitate molecular cloning of resulting A→D fragments (Table 1). *In vivo* using homologous genetic recombination, wild-type loci are then exchanged for recombinant A→D crossover DNA fragments, which yield in-frame, translational deletion alleles of target genes of interest (Methods).

To study the metabolic role of the orthodox respiratory *exo*-hydrogenase activity for H_2_ recycling in diazotrophic culture, *A. caulinodans* haploid strain 66081 carrying an in-frame *hup*Δ*SL2* allele (Table 1), a result of perfect gene-replacement, was constructed by “crossover PCR” mutagenesis [13] (Fig. 1; Methods). To verify its *hupSL* deletion genotype, strain 66081 genomic DNA served as template for diagnostic PCR analysis. Using haploid genomic oligodeoxynucleotides HupSL-Prox and HupSL-Dist (Table 1; Fig. 1) as primer-pair, a single, novel 2.3 kbp DNA fragment was amplified from the 66081 genome, as template, and then sequenced on both strands. Strain 66081 therefore carries the in-frame *hup*Δ*SL*2 allele, arisen by perfect gene-replacement. When tested in chemoautotrophic liquid batch cultures (under 20% H_2_ as sole energy source, 5% CO_2_ as sole C-source, 2% O_2_, bal. N_2_) with ammonium added as sole N-source, whereas parental strain 61305R (virtual wild-type; Methods) grew, strain 66081(*hup*Δ*SL2*) did not. Therefore, HupSL *exo*-hydrogenase activity is required for respiration with exogenous hydrogen. When tested in diazotrophic liquid batch cultures (Methods), strains 61305R and 66081(*hup*Δ*SL2*) both proved fully proficient (able to grow on N_2_ as sole N-source; Nif^+^ phenotype), in comparison to Nif^−^ strains 60107R(*nifA*) and 60035R(*nifD*), both deficient [14]. Strain 66081(*hup*Δ*SL2*) also grew as wild-type when plated on solid, defined medium lacking added-N, thus requiring use of atmospheric N_2_ (Methods). Therefore, orthodox respiratory HupSL *exo*-hydrogenase activity was not material to growth, nor, by presumption, endogenous H_2_ recycling in *A. caulinodans* diazotrophic cultures.

**Table 1:** Bacterial strains and oligodeoxynucleotide primers employed.

***Azorhizobium caulinodans***		
57100	ORS571 wild-type	[1]
60035R	57100 *nifD35R*	[14]
60107R	57100 *nifA107R*	
61305R	57100 Nic^−^ 6-OH-Nic^+^	[29]
66081	61305R *hup*Δ*SL*2	
66132	61305R *hyqΔRI*7	
66203	61305R *hup*Δ*SL*2 *hyqΔRI*7	
66205	57100 *hyqR::uidA^+^ hyq*Δ*BI*, *hyq^+^* merodiploid	
66210	60107R *nifA107R hyqR::uidA^+^ hyq*Δ*BI*, *hyq^+^* merodiploid	
***Escherichia coli***		
MH3000	Δ(*ara-leu*)7697 Δ*lac*(*IPOZY*)X74 *galU* araD139 galK *rpsL ompR101*	[32]
SM10	MM294[::pRP4ΔTn1 Tc^s^] *rec*A Tra(IncP1)^+^ Km^r^	[33]
**Plasmids**		
pSUP202	pBR325 *mob* Ap^r^ Tc^r^ Cm^r^	[33]
pHupΔ*SL*2	pSUP202 *hup*Δ*SL*2	
pHyq ΔRI7	pSUP202 *hyqΔRI*7	
pHyqRU5	pHyqΔRI7 *hyqR::uidA^+^*	
***Oligodeoxynucleotide primers***		
HupSL-Prox	GCCGCAAGGCGCTGCTGA	
HupSL-A	GAAGACGAATTCGCCCGCG	
HupSL-B	*GCCGTCGACGAGCGAGAGGCA*AAGGTCTCGAGGCCGGCCAT	
HupSL-C	*TGCCTCTCGCTCGTCGACGGC*ACCGTGCGCTGAGGGGAGGG	
HupSL-D	CTCGAATTCAAGAGCCATGCC	
HupSL-Dist	ACCTCCGACGGTGCGGTCT	
Hyq-Prox	GAACAGGCGGTGCCAGTTG	
Hyq-A	GCGGAATTCAGGCTGAGGC	
Hyq-B	*GCCGTCGACGAGCGAGAGGCA*GGTGATCATGTGGCCGAAAGA	
Hyq-C	TGCCTCTCGCTCGTCGACGGCCAAAGGGATTAGCCAACACGT	
Hyq-D	CTTCGAATTCGGGCCGC	
Hyq-Dist	CGGACCATCGCTCTGGC	
21-Up	*GCCGTCGACGAGCGAGAGGCA*	
21-Down	*TGCCTCTCGCTCGTCGACGGC*	
UidA-Prox	*CTCGTCGAC *TTACGTCCTGTAGAAACCCCAAC	
UidA-Dist	*GCCGTCGAC *TTGTTTGCCTCCCTGCTGCGG	

### Bioinformatic identification of a novel respiratory *endo*-hydrogenase gene-cluster

**Figure 2:**
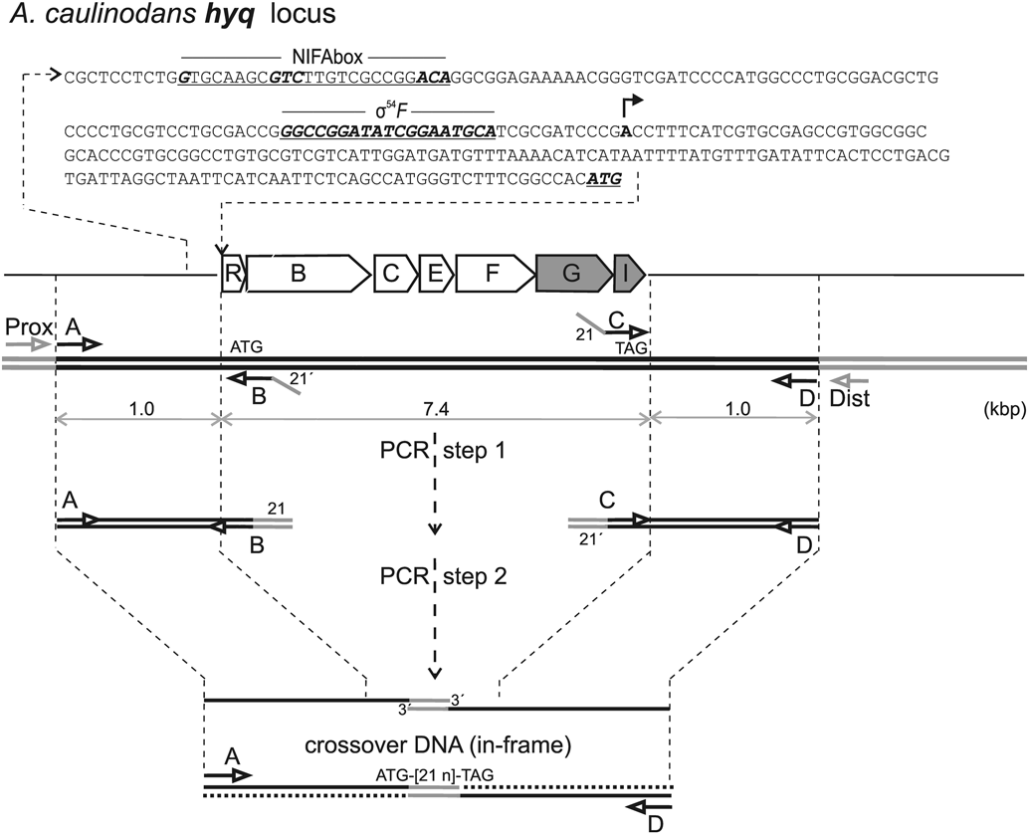
*A. caulinodans hyq* genetic locus and physical map: creation of in-frame, translational fusion deletions. The main line represents the genetic map of the 7-gene *hyq* polycistronic operon (7.4 kbp coding DNA). Superimposed above is the ∼300 np sequence upstream of the *hyqR* start (ATG) codon, presumably comprising the *hyq* control region, which includes a canonical NIFAbox element, facilitating binding of the NIFA transcriptional activator, adjacent to a σ54F-box element, allowing initiation by RNA polymerase·σ54F complex (see Results). For additional details, please refer to Fig 1.

*A. caulinodans* ORS571 genome fragments were then assembled and screened for additional hydrogenase genes. Previously identified, and localized to the same polycistronic operon carrying the *hupSL* genes, were the *hupUV* genes encoding a cytoplasmic sensory hydrogenase activity [15]. From both nucleotide and protein multiple sequence alignments, the *A. caulinodans hupUV* genes proved orthologs of the *R. eutropha hoxBC* genes, which encode a sensory Ni,Fe-hydrogenase coupled to the HoxJ histidine kinase; in *R. eutropha*, this soluble HoxBCJ complex senses H_2_ availability, transactivates *hox* [*hup*] genes in response to H_2_ and is necessarily present only at very low catalytic activity on a per cell basis [16]. Thus, we broadened the hydrogenase search to unlinked loci, initially without benefit of an *A. caulinodans* genomic sequence. Using the BLAT algorithm [17] to search an (∼8 Mbp total) *A. caulinodans* ORS571 shotgun genome sequence dataset (generously provided by B. A. Roe, unpublished results), we assembled several contigs spanning an ∼8 kbp genomic sequence, unlinked to the *hup* operon, but showing homology to Ni,Fe-hydrogenase genes (Fig. 1). From these genome-contigs, we designed synthetic oligodeoxynucleotide primers, carried out PCR amplification and nucleotide sequencing, and assembled the complete sequence for a novel, tightly organized, seven-gene *hyqRBCEFGI* operon (GenBank accession: FJ378904; Fig. 2). In the presumed *hyq* operon, the distal *hyqGI* genes encode a canonical heterodimeric Ni,Fe-hydrogenase. The *hyqBCEF* genes all specify integral-membrane proteins orthologous to the *Escherichia coli hyf* genes, whose syntax in labeling the *A. caulinodans hyq* genes, including *hyqGI*, has thus been conserved. (Note the *A. caulinodans hyq* operon however lacks both *E. coli hyfA* and *hyfD* genes.) The *E. coli hyf* operon encodes hydrogenase-4 [18], an integral-membrane complex representative of the H_2_-evolving or group-4 hydrogenases, previously identified in and restricted to anaerobic bacteria [11].

As well, the group-4 integral-membrane hydrogenases include multiple subunits homologous to those of respiratory NADH : quinone dehydrogenase (NADH-DH), an integral membrane complex whose active site for NADH oxidation faces the cytoplasm [11], [18], [19]. Such homology extends to both NADH-DH L_0_ (integral-membrane) and L_1_ (membrane-associated) sub-complex proteins. By analogy to NADH-DH, and to conceptually distinguish the two *A. caulinodans* cell membrane-associated, respiratory hydrogenases, we therefore denote the presumed HyqBCEFGI complex as *endo*-hydrogenase and the HupSL+cyt*b* complex as *exo*-hydrogenase, so as to distinguish relative orientations of substrate oxidative sites: the former presumably facing the cell interior (cytoplasm), the latter facing the cell exterior (periplasm). As the *A. caulinodans* genome encodes *bona fide* respiratory complex I in an unlinked 15-gene operon (AZC_1667 to AZC_1681) [20], the *hyq* genes and their encoded *endo*-hydrogenase complex, while similar, are indeed structurally and functionally distinct.

### *A. caulinodans* Hyq *endo*-hydrogenase activity facilitates growth in diazotrophic cultures open to the environment

To test metabolic role(s) for the presumptive Hyq *endo*-hydrogenase complex, an *A. caulinodans hyq* operon deletion (*hyqΔRI*7) allele was constructed using crossover PCR, in which the *hyqR* start- and *hyqI* stop-codons were fused in-frame by a 21 bp linker sequence (Fig. 2; Methods). In both strain 61305R(virtual wild-type) and 66081(*hupΔSL2*), the wild-type *hyq* ^+^ operon was then swapped for this *hyqΔRI*7 allele by homologous recombination (Methods). As with strain 66081, the resulting haploid strain 66132 was verified by combined PCR and DNA sequencing analyses. Using haploid genomic Hyq-Prox and Hyq-Dist oligodeoxynucleotides (Table 1; Fig. 2) as primer-pair and strain 66132 genomic DNA as template, a single 2.2 kbp DNA fragment was amplified by PCR and then sequenced on both strands using either Hyq-Prox or Hyq-Dist as DNA sequencing primers. Accordingly, strain 66132 proved a true *hyqΔRI*7 haploid arisen by perfect gene-replacement. Similarly, starting with strain 66081(*hupΔSL2*), strain 66203 proved a true double-mutant haploid, carrying both *hup*Δ*SL*2 and *hyqΔRI*7 alleles.

Growth kinetics of four haploid strains, 61305R and its descendants 66081(*hup*Δ*SL2*), 66132(*hyqΔRI*7) and 66203(*hup*Δ*SL*2, *hyqΔRI*7) were analyzed in liquid batch cultures with defined media. For all strains, batch cultures were started in defined medium with 40 m*M* succinate added as sole C- and energy-source, and 0.5 m*M* ammonium added as N-source. All starter cultures grew exponentially up to viable cell counts of ∼1×10^8^ ml^−1^ at which growth arrested due to ammonium limitation (as when 5 m*M* ammonium was then added, exponential growth rapidly resumed for at least two additional cell doublings). These N-limited, static cultures were one-thousandfold diluted into the same defined growth medium with or without added 5 m*M* ammonium, placed in sealed 30 ml vials, sealed and subcultured with continuous sparging (6 ml min^−1^) using a defined gas mixture (2% O_2_, 5% CO_2_, bal. N_2_). Samples were periodically withdrawn and plated on rich medium for viable cell counts (Methods). Whether in the presence and absence of added ammonium, strains 61305R and 66081(*hup*Δ*SL2*) grew similarly (Table 2). In contrast, while both strains 66132(*hyqΔRI*7) and 66203(*hup*Δ*SL*2, *hyqΔRI*7) grew similarly in the presence of added ammonium, cell doubling-times slowed 21% in the absence of ammonium, relative to strains 61305R and 66081 (Table 2).

**Table 2:** Exponential growth rates of *A caulinodans* strains in diazotrophic liquid batch cultures at 29°C.

Strain	2% O_2_, 5% CO_2_, bal. N_2_ atmosphere (hr)					
	N-source					
	+5 m*M* NH_4_^+^		atm N_2_ only			
	−		−		atm+20% H_2_	
	*t*_D_	*t*_D_(w)/*t*_D_	*t*_D_	*t*_D_(w)/*t*_D_	*t*_D_	*t*_D_(w)/*t*_D_
61305R	2.3*	(1.0)†	7.2*	(1.0)†	4.2*	(1.0)†
66081 *hup*Δ*SL*	2.3	(1.0±.04)	7.2	(1.0±.03)	6.8	(0.62±.02)
66132 *hyqΔRI*	2.4	(0.96±.03)	8.8	(0.82±.02)	5.0	(0.84±.03)
66203 *hup*Δ*SL hyqΔRI*	2.4	(0.95±.05)	8.8	(0.80±.04)	8.8	(0.48±.02)

* doubling-time; representative single experiment.

† doubling-time relative to wild-type (*w*); multiple experiments.

These growth experiments were repeated, except that cultures were sparged with an H_2_-enriched defined gas mixture (2% O_2_, 5% CO_2_, 20% H_2_, bal. N_2_). With the inclusion of H_2_ at saturating metabolic levels in the sparge gas, strains 61305R and 66132(*hyqΔRI*7) when cultured without added ammonium both grew much faster. In one experiment, the diazotrophic cell doubling-time for strain 61305R was cut from 7.2 to 4.16 hr, that for 66132 was cut from 8.8 to 5.0 hr, both in the presence of 20% H_2_ (Table 2). Therefore, while *A. caulinodans* growth rates in liquid diazotrophic cultures were otherwise limited by N_2_ fixation (Table 2), acceleration of oxidative phosphorylation with added 20% H_2_ as respiratory electron-donor also accelerated growth, as is generally characteristic of microaerophilic bacteria [21]. Nevertheless, even with exogenous H_2_ added in sparge gases at levels sufficient to yield maximum growth-rate enhancement, the 21% growth deficit observed for 66132(*hyqΔRI*7) versus parental 61305R persisted. Therefore, cell bioenergetic role(s) for Hyq *endo*-hydrogenase and HupSL *exo*-hydrogenase activities are not entirely synonymous. By analogy to NADH-DH complex activities, Hyq *endo*-hydrogenase activity might also be membrane proton-motive and/or electrogenic, translocating multiple ions such a H^+^, K^+^ and/or Na^+^ (see Discussion).

In contrast, strain 66081(*hup*Δ*SL2*) showed only a slight increase in growth rate in diazotrophic culture, and strain 66203(*hup*Δ*SL*2, *hyqΔRI*7) showed no detectable increase in growth rate, both in response to added 20% H_2_ (Table 2). Therefore in *A. caulinodans*, exogenous H_2_-driven respiration is essentially run by HupSL *exo*-hydrogenase activity, marginally augmented by Hyq *endo*-hydrogenase activity. In contrast, for diazotrophic cultures open to the atmosphere, endogenous H_2_ (produced by Mo-dinitrogenase activity) was exclusively recycled by Hyq *endo*-hydrogenase activity. In enclosed cultures, or when liquid batch diazotrophic cultures open to the atmosphere became sufficiently dense near saturation (>1×10^8^ cells ml^−1^) some amount of endogenous H_2_ recycling by HupSL *exo*-hydrogenase activity was detected (data not presented).

### The *A. caulinodans hyq* operon is strongly and specifically induced in diazotrophic cultures

To assess growth conditions in which the *hyq* operon was genetically expressed, *A. caulinodans* strains using β-glucuronidase activity to report *hyq* operon transcription were constructed. The *E. coli uidA^+^* gene, encoding β-glucuronidase, was amplified by PCR and, using standard *in vitro* molecular cloning techniques, the resulting 1.8 kbp *uidA* ^+^ coding sequence was inserted in-frame into the 21 bp crossover linker sequence of pHyqΔRI7 yielding plasmid pHyqRU5 (Table 1; Methods). Derived from both *A. caulinodans* 57100 and 60107R(*nifA*) as parent, *hyq* merodiploid strains 66205 and 66210, both carrying an upstream in-frame fusion *hyqR*::*uidA* ^+^ *hyq*Δ*BI* operon in tandem with the downstream *hyq* ^+^ operon, were isolated and verified by both PCR and DNA sequencing analyses (Methods). Merodiploid *hyq* reporter strain 66205 was first tested for bacterial colony appearance on solid defined media supplemented with X-Gluc (Methods) as chromogenic β-glucuronidase substrate. When inoculated onto defined medium also supplemented with 5 m*M* ammonium and cultured in fully aerobic conditions, strain 66205 colonies were white, lacking any evidence of β-glucuronidase activity. When the same petri plates were incubated under a reduced O_2_ atmosphere (2% O_2_, 5% CO_2_, bal. N_2_), strain 66205 colonies appeared light-blue, or partially induced. When 0.5 m*M* L-glutamine was added to solid culture medium, 66205 colonies were again completely white when incubated under 2% O_2_ indicating the *hyq* operon was strongly repressed. When 66205 was cultured diazotrophically (in the absence of added ammonium and L-glutamine) under reduced O_2_, colonies were dark blue, indicative of strong *hyq* operon expression.

To obtain quantitative data for both strains 66205 and 66210, liquid batch cultures were pre-grown aerobically in defined medium with 0.5 m*M* ammonium as N-source to cell titers of ∼1×10^8^ ml^−1^ (at which available ammonium was exhausted) and physiologically shifted to diazotrophic culture conditions (Methods) for 12 hr at 29°C. Cells were then harvested and β-glucuronidase specific activities were measured in cell-free extracts (Methods). These results (Table 3) corroborated visual inspections of bacterial plate cultures supplemented with chromogenic X-Gluc. The *hyq* operon was specifically and strongly expressed in diazotrophic culture but strongly repressed either in the presence of added ammonium under air or in the presence of added 0.5 m*M* L-glutamine under reduced O_2_. As strain 66210 was only weakly induced (Table 3), *hyq* operon induction specifically required NIFA transcriptional activation.

**Table 3:** *A caulinodans hyq* operon expression; P*hyqR* β-glucuronidase reporter activity.

Strain	Atmosphere	N-source(s)		
		N_2_ only	+5 m*M* NH_4_^+^	+5 m*M* NH_4_^+^
		(atm = 78+%)		+05 m*M* L-glutamine
66205	2% O_2_	1410±200	220±35	<10
	21% O_2_		<10	<10
66210 *nifA*	2% O_2_	40±10	40±10	<10
	21% O_2_		<10	<10

a nmol 5-bromo-4-chloro-3-indole min^−1^ mg protein^−1^.

When the presumed *hyq* operon control region (immediately upstream from the *hyqR* coding sequence) was analyzed, the genetic signatures of an orthodox *A. caulinodans nif* operon were apparent (Fig. 2). A NifAbox element, serving as *cis*-acting site for NIFA transactivation, and a σ54_N_ site, serving as *cis*-acting site for RNA polymerase complexed with σ54_N_ initiation factor, were both present and strategically positioned [22]. Thus, the *A. caulinodans hyq* operon control region likely binds NIFA, which activates *hyq* transcription via RNA polymerase · σ54_N_ in response to both limiting physiological O_2_ and absence of fixed-N, as is observed for *nifA* autoregulation [22]. Accordingly, the *A. caulinodans hyq* operon is constituent of the *nif* regulon.

## Discussion

In rhizobia, obligate oxidative bacteria, orthodox respiratory Ni,Fe-hydrogenase is encoded by contiguous *hupSL* genes. (In other obligate oxidative bacteria, orthologous gene assignments are variant, *e.g. hoxKG* in *R. eutropha* [12]). Notably, the Ni,Fe-catalytic center of this conserved respiratory hydrogenase complex is periplasmic-oriented, *i.e. exo*-hydrogenase. Indeed, orthologous rhizobial HupS and *R. eutropha* HoxK encoded FeS-center subunits possess a periplasmic export (RRxFxK) signal peptide motif [11]. Typical of H_2_-recycling rhizobia, the *A. caulinodans exo*-hydrogenase encoding genomic locus comprises a 21 kbp contiguous set of highly-conserved genes, among them *hupSL* [15] (Fig. 1). This respiratory hydrogenase activity is obviously adapted for use of exogenous H_2_.

Archetypal for the group-4 hydrogenases is *E. coli* hydrogenase-3, encoded by *hycGE*. This heterodimeric Ni,Fe-hydrogenase anchors an integral-membrane formate–hydrogen lyase complex, oxidizing formate to CO_2_ and reducing 2H^+^ to H_2_, all cell-internal, under strict, fermentative conditions [19]. In *E. coli*, a second group-4 hydrogenase (hydrogenase-4), encoded by the *hyf* operon, seems coupled to yet another fermentative formate dehydrogenase activity [18]. In *Rhodospirillum rubrum* a distinct, but related, group-4 hydrogenase activity is coupled to CO-dehydrogenase activity [23], [24]. In all cases these group-4 hydrogenases are active under anaerobic, strictly fermentative physiological conditions and so have been termed H_2_-evolving, simultaneously oxidizing either formate or CO to yield CO_2_ and reducing H^+^-ions to yield H_2_ (gas), all as fermentative end-products.

From multiple protein sequence alignments, the *A. caulinodans* HyqBCEFGI hydrogenase is a constituent member of the group-4 hydrogenases. However, as *A. caulinodans*, like all rhizobia, is an obligate oxidative (aerobic) bacterium and does not ferment, any metabolic role for H_2_ evolution is not obvious. Indeed, we have identified unlinked *A. caulinodans* genes encoding both formate- and CO-dehydrogenase activities; the former are orthologous to the aerobic, respiratory formate dehydrogenase of facultative bacteria such as *E. coli*; the CO-dehydrogenase genes are orthologous to the soluble, NAD-linked activities of obligate aerobic bacteria (data not presented).

From bacterial genome searches, orthologous *hyq* operons are evident in two additional rhizobial species, *R. leguminosarum* and *B. japonicum* both previously classified phenotypically as H_2_ recyclers [9], [25]. In the non-symbiotic but very closely related species *Xanthobacter autotrophicus* Py2 [26], an orthologous *hyq* operon is also present, as is the complete *nif* regulon, implying *X. autotrophicus* Py2 is also diazotrophic. All such bacteria carrying the *hyq* operon are obligate oxidative, in which any H_2_-evolving hydrogenase activities would seem not only superfluous but antithetical.

Metabolic roles for the group-4 hydrogenases are not definitive. All show integral-membrane components with homology to NADH : quinone dehydrogenase (respiratory complex I), which functions as unidirectional NADH oxidant and membrane quinone pool reductant [11], [18], [19]. Included in this homology are the heterodimeric Ni,Fe-hydrogenase subunits of group-4 hydrogenases (the *A. caulinodans* HyqGI proteins) which are closely related to the 49 kDa (Nqo4) and 20 kDa (Nqo6) subunits of the *Thermus thermophilus* (hyperthermophile) respiratory NADH-DH L_1_ sub-complex, whose crystal structure has been solved by X-ray diffraction at atomic resolution [27]. By structural analogy and genetic homology to the NADH-DH L_1_ sub-complex then, the homologous HyqGI heterodimeric *endo*-hydrogenase, with its active site facing cell-internally, likely interacts with the integral-membrane, L_0_-homologous HyqBCEF sub-complex and together function as H_2_ oxidant and membrane quinone reductant. Like both NADH-DH complex and *E. coli* hydrogenase-4, *A. caulinodans* Hyq *endo*-hydrogenase activity is presumably proton-motive, energy-conserving [18] and thus likely drives aerobic respiration. From multiple protein alignments including sequences identified in the four aerobic bacteria (*A. caulinodans*, *B. japonicum*, *R. leguminosarum*, *X. autotrophicus*), together with the *E. coli* HyfGI proteins, the *endo*-hydrogenase peripheral HyqG large-subunit carries the Ni,Fe-hydrogenase catalytic center and the HyqI small-subunit carries the (N2) proximal 4Fe–4S center as likely electron-donors to membrane-bound quinones.

Given this inferred organization and integral-membrane orientation of the Hyq *endo*-hydrogenase complex (for which we as yet lack direct experimental evidence), one implication is obvious: the Hyq *endo*-hydrogenase might physically interact with Mo-dinitrogenase so as to channel evolved H_2_ as substrate for membrane-driven respiration and oxidative phosphorylation. Coupled respiration would allow quantitative recovery of ATP consumed by Mo-dinitrogenase complex activity in H_2_ synthesis (and activation of N_2_ reduction to ammonium) [3], [4]. The group-4 hydrogenases of anaerobes, while quite possibly active *in vivo* in H_2_ evolution during strictly fermentative metabolism, nevertheless remain capable of H_2_ oxidation, albeit slowly [11]. Because Mo-dinitrogenase complex activity has exceedingly slow *in vivo* turnover (<10 sec^−1^), any directly coupled Hyq *endo*-hydrogenase H_2_ oxidizing activity might operate at correspondingly very slow rates *in vivo*.

H_2_-oxidative *endo*-hydrogenase activity necessitates that H^+^ ions be membrane-translocated else deplete the cell membrane proton-motive force. Any *endo*-hydrogenase driven, vector H^+^ translocation, an energy-requiring process, would be necessarily slow by comparison with *exo*-hydrogenase activity, uncoupled from H^+^ translocation, and thus relatively fast. (In the latter case, as H^+^ ions are produced external to the cell membrane, they in principle contribute directly to the cell membrane proton-motive force.) Thus, *exo*-hydrogenase activity is kinetically preferred as agent for exogenous H_2_ oxidation. By contrast, *endo*-hydrogenase activity, via metabolic channeling, might confer an increased efficiency of endogenous H_2_ recycling, thus mitigating energy loss, were such H_2_ to escape to the environment by simple diffusion.

Hydrogen has elicited considerable interest as potential renewable energy source for human civilization. If hydrogen is to be produced at scale as part of a sustainable cycle, external energy source(s) are then required. Solar energy represents an obvious energy coupling source, in principle allowing photoelectron transport and H_2_-evolving hydrogenase activities to operate as an integrated biocatalytic process in photosynthetic membranes. Accordingly, the reversible group-4 hydrogenases, such as the *A. caulinodans* Hyq *endo*-hydrogenase, offer particular promise as biocatalytic agents for hydrogen production and/or consumption.

## Methods

### Bacterial strains and culture conditions

*Azorhizobium caulinodans* ORS571 wild-type (strain 57100), originally isolated from *Sesbania rostrata* stem-nodules [1], was cultured in both rich (SYPC) and miminal, defined media as previously described [14]. As 57100 wild-type is NAD auxotrophic, defined growth media must be supplemented with nicotinate (or similar) as precursor. However, nicotinate serves strain 57100 as both anabolic (for NAD production) and catabolic (as both utilizable C- and N-source) supplement. When strain 57100 is cultured in media with limiting primary C- and/or N-sources, nicotinate is rapidly catabolized and exhausted cultures quickly become NAD- limited for growth [28]. Accordingly, to eliminate nicotinate catabolism as a metabolic variable, all experiments reported herein employ *A. caulinodans* 61305R, a 57100 derivative carrying an IS*50*R insertion in the (catabolic) nicotinate hydroxylase structural gene, as “virtual” wild-type; 61305R only uses nicotinate as anabolic substrate and thus requires minimal (1 µ*M*) nicotinate supplementation in all defined media [29].

### Genetic constructions

#### *A. caulinodans* in-frame translational fusion mutants

Precise, in-frame deletion mutagenesis of the *A. caulinodans hupSL* genes was carried out by “crossover PCR” as previously described [13]. In the first step, separate ∼1 kbp genomic fragments immediately proximal to *hupS* and distal to *hupL* coding sequences were PCR amplified [13]. These two, ∼1 kbp amplified genomic fragments shared an artificial, complementary “crossover” sequence introduced by 21 bp extension of PCR primers “B” and “C” (Fig. 1). In a second-round PCR, the two amplified DNA fragments were purified, mixed, and used as combination primer-template. A ∼2 kbp DNA fragment was then produced when non-homologous template strands annealed via complementary 21 bp extensions; when an upstream coding-strand annealed to a downstream non-coding strand via the 21 bp crossover extension, 3′-ends on both annealed strands were extended by thermostable DNA polymerase yielding a contiguous ∼2 kbp DNA fragment in which the 21 bp crossover sequence fused the ∼1 kbp upstream and downstream sequences. In this second-round PCR, terminal “A” and “D” oligodeoxynucleotides (Fig. 1) were also included as primers such that, by standard recursive PCR, this ∼2 kbp crossover DNA fragment was further amplified. By design, the 21 bp crossover within the ∼2 kbp DNA fragment fuses in-frame an upstream target gene's translational “start” codon with a downstream target gene's “stop” codon yielding a translational (*e.g.*, *hup*Δ*SL*) fusion (Fig. 1).

The PCR amplified, crossover DNA fragment carrying the in-frame ∼2 kbp *hup*Δ*SL*2 fusion allele was verified by DNA sequencing and introduced into the *Eco*RI site of plasmid pSUP202 (Table 1) by standard molecular cloning; *E. coli* strain MH3000 (Table 1) was subject to electroporation with recombinant plasmids, and transformed bacterial colonies were selected for tetracycline (Tc) resistance. In this manner, recombinant plasmid pHupΔ*SL*2 was identified (Table 1), purified, and reintroduced by electroporation into *E. coli* SM10 (Table 1), proficient as donor for bacterial conjugation, again selecting for Tc-resistance. To allow plasmid conjugal transfer, *E. coli* SM10/pHupΔ*SL*2 as donor was mixed with *A. caulinodans* 61305R as recipient and plated overnight on SYPC solid medium at 37°C. Conjugal cell mixtures were then selectively plated on solid ORSMM (to counter-select *E. coli*) supplemented with Tc (10 µg/ml) at 37°C. As parental plasmid pSUP202 cannot stably replicate in *A. caulinodans*, transconjugants that are stably Tc-resistant arise after homologous, single recombination events in which the entire plasmid and the target genome are cointegrated [22]. Accordingly, *A. caulinodans* 61305R *hupSL* merodiploids were then isolated and confirmed by PCR and DNA sequencing analyses; such strains carried both genomic *hupS^+^L^+^* and *hup*Δ*SL2* alleles bridged by the integrated SUP202 sequence. To then isolate haploid gene-replacement strains, merodiploids were subcultured absent Tc selection in rich GYPC liquid medium and then plated with Tc added at very low (0.125 µg/ml) levels sufficient to 50% inhibit growth of parental wild-type. Pinpoint colonies were identified, retested, and a Tc-sensitive phenotype verified. These putative haploid derivatives arose by a second, single homologous recombination (disintegration) event within the merodiploid, segregating the *hupSL* alleles. By PCR analysis with Hup-Prox and Hup-Dist as oligodeoxynucleotide primer-pair (Table 1; Fig. 1), resulting haploid strains showed either *hupS^+^L^+^* or *hup*Δ*SL2* alleles.

Similarly, a haploid 61305R derivative carrying a complete *hyqRBCEFGI* in-frame deletion allele was isolated using the same crossover PCR technique. Recombinant plasmid pHyqΔRI7 carried a ∼2 kbp *hyqΔRI*7 allele in which the identical 21 bp linker fused in-frame the *hyqR* “start” codon with the *hyqI* “stop” codon (Table 1; Fig. 2). After gene replacement, haploid strain 66132 carrying the *hyqΔRI7* allele was isolated and verified by PCR and DNA sequencing analyses.

#### *A. caulinodans* Hyq transcriptional reporter strains

To construct *hyq* merodiploid transcriptional reporter strains, a 1.8 kbp fragment carrying the *E. coli uidA^+^* coding sequence was amplified by PCR using synthetic oligodeoxynucleotide primers extended with 6 bp *Sal*I endonuclease recognition sequences (Table 1). As the 21 bp linker sequence used to construct in-frame translational fusions includes a *Sal*I recognition sequence, plasmid pHyqΔRI7 was partially digested with *Sal*I endonuclease, a 9.8 kbp DNA fragment was isolated, mixed with *Sal*I digested, amplified 1.8 kbp *uidA^+^* DNA fragment and recombinant plasmids were recovered by standard molecular cloning techniques. After electroporation of *E. coli* MH3000, and selection for Tc-resistance, *uidA* ^+^ recombinant plasmids were identified by plating candidate strains on minimal media supplemented with (0.1 mg ml^−1^) 5-bromo-4-chloro-3-indolyl-β-D-glucuronide (XGluc), a chromogenic β-glucuronidase substrate, and screening for blue colonies. From PCR and DNA sequencing analysis, recombinant plasmid pHyqRU5 carrying the *hyqR::uidA^+^* in-frame translational fusion allele was isolated (Table 1). Plasmid pHyqRU5 was introduced to E. coli SM10, and this strain was employed as conjugal donor with *A. caulinodans* 61305R and 60107R, and Tc-resistant derivatives were selected and isolated. Merodiploid strains 66205 and 66210 (Table 1) carrying both upstream *hyqR::uidA^+^ hyq*Δ*BI* and downstream *hyq^+^* operon were identified and verified by PCR and DNA sequencing analyses.

#### Physiological growth and β-glucuronidase activity measurements

Starter cultures of *A. caulinodans* strain 61305R and its derivatives were aerobically cultured in minimal defined NIF liquid medium [14] supplemented with: ammonium (0.5 mM) as sole, limiting N-source and 1 u*M* nicotinate at 37°C until growth arrested (cell densities ∼1×10^8^ cells ml^−1^). For kinetic measurements of diazotrophy, arrested starter cultures were diluted one-thousandfold in NIF medium (with 1 u*M* added nicotinate) into 30 ml serum vials, sealed with silicone rubber septa, sparged continuously (6 ml min^−1^) with defined gas mixtures (*e.g.* 2% O_2_, 5% CO_2_, bal. N_2_), and incubated at 29°C. At least three times per cell-doubling period, culture samples were removed, serially diluted, plated on rich GYPC medium [14], incubated 48 hr at 37°C, and colonies were counted, in triplicate. β-glucuronidase activity was measured with as chromogenic substrate 5-bromo-4-chloro-3-indolyl-β-D-glucuronide (X-Gluc) [30]; total protein concentrations were determined in a folin phenol reagent assay [31]. All induction experiments were conducted in triplicate and were repeated until the standard error in β-GUS activities was below 15%.
